# Emerging role of senescent microglia in brain aging-related neurodegenerative diseases

**DOI:** 10.1186/s40035-024-00402-3

**Published:** 2024-02-20

**Authors:** Chan Rim, Min-Jung You, Minyeop Nahm, Min-Soo Kwon

**Affiliations:** 1 Department of Pharmacology, Research Institute for Basic Medical Science, School of Medicine, CHA University, CHA Bio Complex, 335 Pangyo, Bundang-gu, Seongnam-si, Gyeonggi-do 13488 Republic of Korea; 2 Brainimmunex Inc., 26 Yatap-ro, Bundang-gu, Seongnam-si, Gyeonggi-do 13522 Republic of Korea; 3 Dementia Research Group, Korea Brain Research Institute, Daegu, Republic of Korea

**Keywords:** Senescent microglia, Rejuvenation, Neurodegenerative diseases, Brain aging

## Abstract

Brain aging is a recognized risk factor for neurodegenerative diseases like Alzheimer's disease, Parkinson's disease, and amyotrophic lateral sclerosis (ALS, Lou Gehrig's disease), but the intricate interplay between brain aging and the pathogenesis of these conditions remains inadequately understood. Cellular senescence is considered to contribute to cellular dysfunction and inflammaging. According to the threshold theory of senescent cell accumulation, the vulnerability to neurodegenerative diseases is associated with the rates of senescent cell generation and clearance within the brain. Given the role of microglia in eliminating senescent cells, the accumulation of senescent microglia may lead to the acceleration of brain aging, contributing to inflammaging and increased vulnerability to neurodegenerative diseases. In this review, we propose the idea that the senescence of microglia, which is notably vulnerable to aging, could potentially serve as a central catalyst in the progression of neurodegenerative diseases. The senescent microglia are emerging as a promising target for mitigating neurodegenerative diseases.

## Introduction

It is clear that addressing ‘aging’ presents an appealing approach to treating neurodegenerative diseases [1]. Advances in computer and biological sciences, particularly in next-generation sequencing technologies and machine learning, have facilitated the establishment of cell-type-specific aging clocks. These clocks utilize transcriptomic and phenotypical data from specific cells to predict biological aging [2]. In alignment with the aging clocks, the Mouse Aging Cell Atlas, also known as the Tabula Muris Senis, has been developed at a single-cell resolution to comprehend the cellular characteristics of the entire mouse organ [3]. Human plasma proteome analysis has revealed organ-specific aging differences using machine learning models, indicating that most human aging may be initiated by acceleration in a single organ [4]. Particularly, accelerated brain and vascular aging predict Alzheimer’s disease (AD) progression [4]. Although previous studies have provided valuable insights into the cellular landscape and molecular changes associated with aging in mice, our understanding of the cellular functions and metabolism contributing to brain aging phenotypes remains limited. Recent studies propose that senescent microglia, rather than reactive microglia, could be a novel therapeutic target in neurodegenerative diseases [5–7]. In this paper, we elaborate on how senescent microglia have emerged as a promising therapeutic target for neurodegenerative diseases, building on the significant advances in microglial research over the past decade.

## The beginning of aging: cellular senescence

Cellular senescence refers to a state in which cells undergo irreversible cell cycle arrest and other cellular/molecular changes that render them dysfunctional. Senescent cells are different from cells within an aged organ (aged cells) [8]. This state is commonly triggered by DNA damage or other cellular insults [9]. Cellular senescence is characterized by alterations in gene expression, physiological function, and cell morphology [10]. Various stressors can lead to cellular senescence, including replicative senescence caused by long-term proliferation, DNA damage-induced senescence, oxidative stress-induced senescence, oncogene-induced senescence, nuclear barrier-induced senescence, and reciprocally induced cellular senescence [11, 12]. Senescent cells have been identified in pathological conditions such as cancer [13], chronic inflammation [14], and tissue fibrosis [15], as well as in aged organisms and during developmental stages of organisms [16].

DNA damage plays a central role in the aging process [17]. Excessive DNA damage and insufficient DNA damage repair contribute to cellular aging [18]. Endogenous and exogenous factors that induce DNA damage, such as reactive oxygen species (ROS), replication errors, chemicals, and UV radiation, accumulate over time [19]. This accumulation of DNA damage may result in the production of atypical proteins, triggering apoptosis or cell cycle arrest [20, 21]. DNA repair that counters the consequences of DNA damage accumulation and cellular senescence, relies heavily on ATP, leading to alterations in energy production, another hallmark of cellular senescence [22, 23]. In response to an increased demand for energy, senescent cells alter their metabolic states, resulting in inefficient ATP production and lipid droplet accumulation [24]. Furthermore, depending on the specific cell type and environment, senescent cells often exhibit a senescence-associated secretory phenotype (SASP), which entails metabolic alterations and the secretion of various molecules, such as matrix metalloproteinases (MMPs) that can modify their immediate surroundings [25]. The SASP not only recruits immune cells to eliminate senescent cells, but also contributes to inflammation and tissue remodeling [26]. Additionally, the SASP plays a crucial role in senescence transmission through paracrine signaling to neighboring cells [27], creating a chronic, low-grade inflammatory environment, known as inflammaging [28, 29].

## Senescent microglia as an emerging player in brain aging

G1 phase arrest is a key characteristic of cellular senescence [30]. While it has been well-established in proliferating cells, the applicability of G1 phase arrest to post-mitotic cells that have already undergone differentiation is still under investigation and lacks a definitive standard [31]. However, depending on aging or exposure to oxidative stress and damage, post-mitotic cells such as neurons can exhibit signs of senescence without undergoing neurodegeneration. Neurons, like other cells, express senescence markers such as lipofuscin, which accumulates into autofluorescent aggregates with age [32]. Furthermore, neurons exhibit DNA damage and increased secretion of SASP components [25]. In addition, the expression of p21, a protein involved in suppressing proliferation, is increased in neurons experiencing cellular senescence [33]. Interestingly, at least transient upregulation of p21 in neurons appears to be independent of the cell cycle, and is possibly linked to the formation of ROS and oxidative stress [33]. Glial cells, including astrocytes, microglia, and oligodendrocytes, also exhibit post-mitotic features as they acquire a mature phenotype in a healthy state. These cells are long-lived and exhibit senescence-associated features such as lipofuscin accumulation, DNA damage, loss of lamin B1, increased activity of senescence-associated β-galactosidase (SA-β-gal), lysosomal dysfunction, and SASP, both in vitro and in aged mouse brains [34–36].

Unlike neurons, astrocytes and oligodendrocytes, which can be replenished through neurogenesis and gliogenesis in adulthood, microglia are unique in that they originate from the yolk sac, not the neuroectoderm [37]. During development, microglia progenitor cells, derived from hematopoietic stem cells in the yolk sac, migrate into the brain parenchyma before the formation of the blood–brain barrier (BBB) [38]. Once settled in the brain, they mature and persist throughout an individual’s lifetime [39]. Although the loss of microglial cells might be replaced by the proliferation of resident microglia, their capacity for repopulation is limited [40]. Reports suggest that the infiltrating monocytes can fill the niches left by lost microglia [41]; however, primary replenishment is generally expected to come from existing resident microglia, although this niche replenishment depends on the methods of microglial depletion [42]. Studies have shown that the repopulated microglia can have different transcriptomes compared to the original microglia derived from the yolk sac [42, 43]. As a result, studies using single-cell tracking in mice have revealed that microglia originating from the yolk sac have a relatively long lifespan, averaging ~ 15 months, with some cells having the ability to persist throughout an individual's entire lifespan [39, 44]. This extended lifespan of microglia from the yolk sac might have significant implications, especially considering their susceptibility to various senescence-inducing factors such as DNA damage, ROS, replicative stress, protein aggregates, and glucocorticoids [45–48].

The accumulation of senescent microglia, along with their altered immune functions and interactions with other brain cells, could potentially play a critical role in the pathogenesis of neurodegenerative conditions [49]. Thus, the unique origin and characteristics of yolk sac-derived microglia, which exhibit a limited repopulating capacity, suggest that they have an intrinsic connection to their senescence and might contribute significantly to the pathogenesis of aging-related neurodegenerative diseases [50–53].

Recent spatiotemporal RNA-seq findings have revealed accelerated aging in glial cells, particularly in white matter, identifying microglia as a potential major contributor to brain aging, as determined through Bulk-seq Common Aging Score calculations [50]. This suggests that microglia might be the most vulnerable brain cells to aging. According to the threshold theory of senescent cell accumulation [54], when the number of senescent cells in the body rises to surpass a threshold, the immune system and other organs would be more prone to aging-related diseases [55]. In other words, once a certain number of senescent cells accumulate, they may lose the ability to function normally. SASP factors from a small population of senescent cells (transient SASPs) are beneficial for senescent cell clearance [56]. However, over time, the accumulated senescent cells show persistent SASP, accelerating cellular senescence through inflammaging [56]. Although microglia make up only 5%–10% of the total brain cells, their diverse roles suggest that a high proportion of senescent microglia may initiate brain aging and lead to its propagation. Microglia also exhibit spatial and temporal heterogeneity, which is relevant to the regional sensitivities to aging [57, 58]. To date, there has been no report explaining why microglia in white matter, which consists of abundant myelin, appear to be susceptible to the effects of aging. Nonetheless, we have a few speculations. First, microglia are responsible for phagocytosing and clearing debris to maintain CNS homeostasis [59]. Accumulation of myelin debris with abundant cholesterol may pose a chronic phagocytic challenge to microglia, affecting intracellular cholesterol metabolism via their receptor triggering receptor expressed on myeloid cell 2 (TREM2) [60]. In fact, TREM2 deficiency results in fewer senescent microglia in AD mice, suggesting that the senescence of microglia is TREM2-dependent [61]. Lipids derived from persistent demyelinating processes can trigger lysosomal dysfunction, leading to the generation of lipid droplet-accumulating microglia (LDAM), as observed in the aged brain. However, it remains to be confirmed whether the LDAM share a similar transcriptome with senescent microglia [62, 63]. Deficiency in *Grn*, a risk gene for frontotemporal dementia, leads to severe accumulation of lipid droplets in microglia in mice. However, further studies are needed to determine whether LDAM and *Grn*^−/−^ microglia share senescent characteristics [63].

Regarding senescence spreading, senescent cells contribute to inflammation and promote senescence of neighboring cells through SASP. The SASP-related secretory molecules include various pro-inflammatory and anti-inflammatory cytokines, chemokines, and other molecules secreted in response to cellular senescence. There is no single representative molecule for the SASP, and the composition of SASP varies depending on the specific type of senescent cell. However, it typically includes interleukin (IL)-6, IL-8, MCP-1 (monocyte chemoattractant protein-1), interferon-gamma (IFN-γ), MMP-1, prostaglandin E2 (PGE_2_), ROS, and other factors that can enhance cellular senescence in an autocrine manner and propagate senescence to surrounding cells in a paracrine manner [25]. A significant mechanism by which SASP contributes to senescence is through the induction of secondary senescence of neighboring cells [64]. This phenomenon can have profound implications for tissue and organismal aging [64, 65]. Interestingly, an analysis of SASP reveals that many of its components are markers associated with the neuroinflammatory response, with a majority being substances secreted in high amounts by microglia [51]. The accumulation of local senescent microglia might induce paracrine senescence in neighboring cells (including other glial cells and neurons) through SASP-related secretory molecules, leading to the aging of the entire brain [52, 66].

## Senescent microglia: a universal target in neurodegenerative diseases

The term “dystrophic microglia” denotes structural changes in senescent microglia with aging [52, 67–70]. These alterations have been observed in proximity to tau and amyloid pathologies within the brains of AD patients, as well as near sites of Lewy bodies in individuals with dementia [7, 71]. Notably, inflammatory microglia occur during the early stages of AD. As this activation subsides, microglia transit to a senescent or dystrophic state, becoming less responsive to stimuli in later stages [72]. Histopathological examinations involving 19 instances of AD pathology have suggested that microglial degeneration due to aging might contribute more significantly than microglial activation to the onset and progression of AD [73].

The concept of the disease-associated microglial (DAM) phenotype around amyloid β (Aβ) was first described as a universal immune sensor in the context of AD, brain aging, and ALS [74]. In that paper, the DAM signature, induced by factors associated with neurodegeneration such as apoptotic neurons, myelin debris, and aggregated proteins, is characterized by upregulation of genes involved in phagocytic function and lipid metabolism in a TREM2-dependent manner, followed by TREM2-independent downregulation of the microglial inhibitory-check pathway [75]. Because DAM are phagocytic cells, they have been expected to be beneficial in AD therapeutics. The microglial neurodegenerative phenotype (MGnD) is another phenotype that is characterized by a specific TREM2-apolipoprotein E (ApoE)-dependent molecular signature surrounding neuritic Aβ plaques. MGnD microglia are dysfunctional microglia observed in AD and ALS [76]. Thus, MGnD microglia were initially expected to have a detrimental effect in neurodegenerative diseases. However, both DAM and MGnD surrounding Aβ have been discovered by single-cell analysis, and DAM might evolve into or overlap with MGnD functions [77]. Nevertheless, it appears evident that the TREM2-ApoE pathway plays a crucial role in microglial function and phenotype in neurodegenerative diseases.

Regarding the TREM2 pathway and microglial senescence, microglial TREM2 can regulate metabolism via the mammalian target of rapamycin (mTOR) signaling, which is a key regulator in cellular senescence [78]. Compared to acute Aβ exposure, persistent exposure to Aβ results in compromised microglial function, marked by immune tolerance and metabolic deficits (defective glycolytic metabolism), including suppressed mTOR phosphorylation [79], although it has not been confirmed whether microglia exposed to chronic Aβ exhibit a senescent phenotype. A report indicates that the senescent microglia increase lactic acid production, suggesting enhanced glycolysis [80]. In human cardiac progenitor cells, pharmacological inhibition of mTOR attenuates replicative senescence [81]. Thus, the regulation of mTOR and the type of metabolic pathway appear to be determined in a cell type- and context-dependent manner in senescent cells.

Chronic tau exposure also induces microglial senescence, characterized by cell cycle arrest, DNA damage, loss of lamin B1, impaired tau clearance, and formation of a SASP [82]. TDP-43 aggregates, present in 97% of ALS patients [83], interact with microglial TREM2 and consequently increase their phagocytosis and clearance by microglia [84]. TREM2 deficiency impairs the phagocytosis of pathological TDP-43 [85]. Similar to Aβ and tau, the phagocytosis of TDP-43 aggregates by microglia typically results in the activation of the microglial NLRP3 inflammasome and upregulation of proinflammatory markers [86], all contributing to the neurotoxic effects on motor neurons. However, the phenomenon of microglial senescence due to prolonged exposure to TDP-43 aggregates has not been thoroughly investigated.

**Fig. 1:**
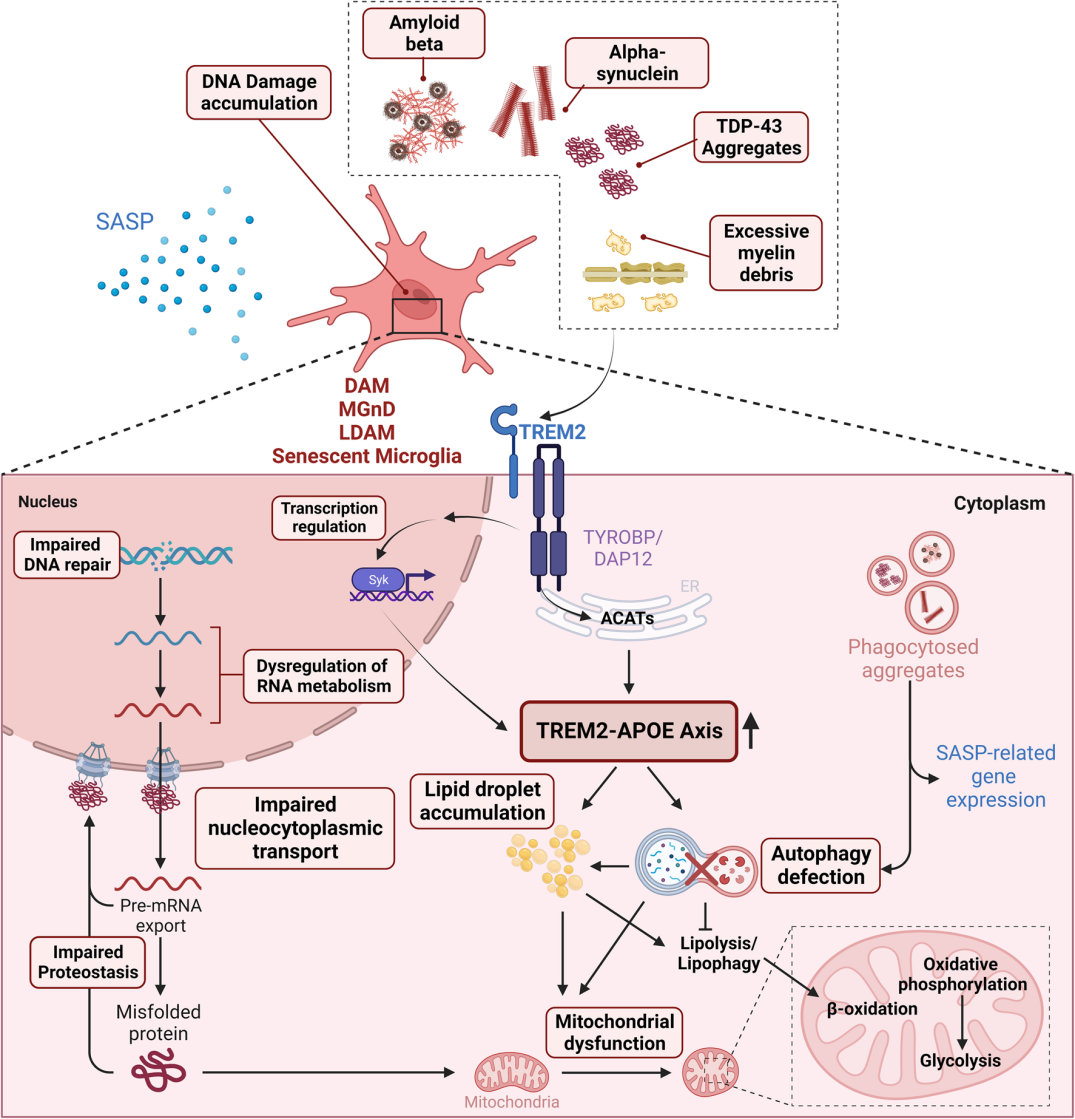
Proposed mechanism of the TREM2-APOE axis-mediated microglial senescence among previously reported microglial phenotypes. Accumulated DNA damage can dysregulate RNA metabolism and proteostasis, leading to accumulation of cytoplasmic aggregates and disruption of nucleocytoplasmic transport. Furthermore, chronic exposure to extracellular protein aggregates induces autophagy, mitochondrial dysfunction, and secretion of SASPs, with a primary focus on the TREM2-APOE axis. Created with Biorender.com

Interestingly, senescent microglia induced by replicative stress are enriched in DAM signatures [45]. In addition, this study confirmed that microglia surrounding amyloid plaques express relatively higher levels of senescence markers, and DAMs also exhibit senescence characteristics. Moreover, the research group that reported DAM found that TREM2-null AD mice exhibited lower accumulation of senescent microglia compared to the AD mice with intact TREM2 [61]. This suggests that the senescent microglia are likely dependent on TREM2 and APOE, as mentioned earlier. If this is the case, a key question arises: how do DAMs and senescent microglia, both stemming from a TREM2-dependent pathway, exhibit opposing functions? This may explain why therapeutics designed to activate TREM2 have shown limited effects in preclinical studies [87]. TREM2 play diverse roles in lipid sensing for survival, eliminating toxic protein aggregates, and clearing myelin debris in microglia [85, 88–90]. When microglia are subjected to larger quantities and chronic exposure to these ligands, they may become senescent via TREM2 activation [61]. This possible mechanism is described in Fig. 1.

**Fig. 2:**
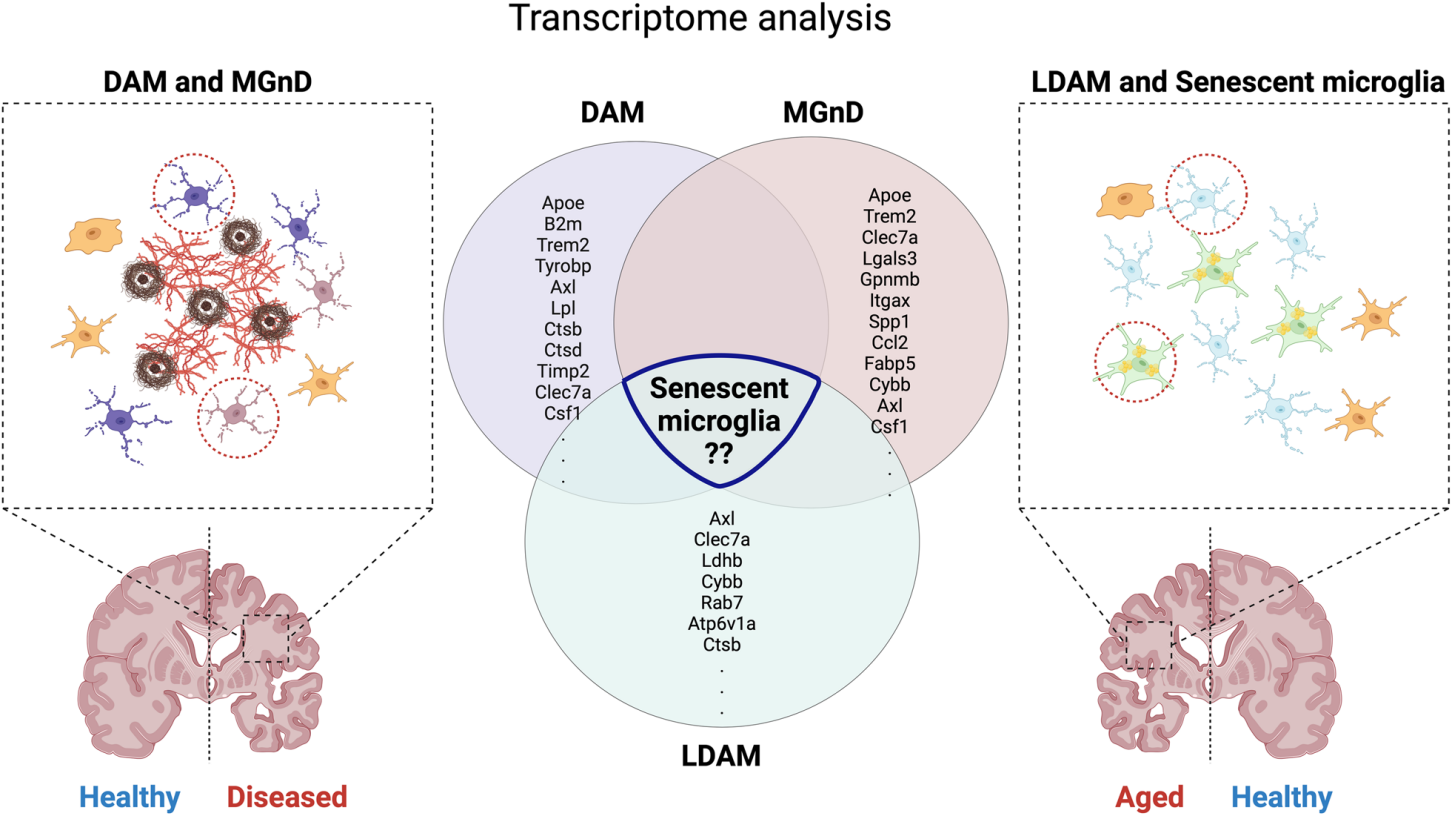
Previously reported microglial subpopulations may share features with senescent microglia. Microglia in the neurodegenerative brain can be classified into distinct phenotypes: disease-associated microglia (DAM) and neurodegenerative microglia (MGnD). In the aged brain, microglia exhibit senescent features and accumulate lipid droplets (LDAM). Previous studies suggest that senescent microglia contribute to the pathophysiology of neurodegeneration, as the gene expression patterns of previously reported microglia overlap with those of senescent microglia. Created with Biorender.com

A recent study also revealed that the loss of TREM2 enhances the expression of genes associated with a homeostatic state of microglia, while microglia derived from granulin knockout (*Grn*^−/−^) mice exhibit reciprocal activation of the MGnD molecular signature and suppression of genes characteristic of homeostatic microglia [91]. Both scenarios result in neurodegeneration through mechanisms involving loss of function. Given that the *Grn*^−/−^ mice can induce LDAM, LDAM might share signatures with MGnD. On the other hand, loss of TREM2 increases the homeostatic state of microglia. Since senescent microglia are TREM2-dependent [91], loss of TREM2 might contribute to the elimination of senescent microglia, leading to an increased population of homeostatic microglia [89]. To address this issue, further transcriptomic comparison studies among DAM, MGnD, LDAM, and senescent microglia are needed (Fig. 2).

In the context of mutant superoxide dismutase 1 (mSOD1) transgenic mice, an established mouse model for familial ALS, studies have demonstrated that microglia play a role in neuroinflammation [92]. However, we need to reinterpret the findings because new microglia-specific markers, which distinguish resident microglia from peripheral monocytes entering the brain, have been identified. Using the new microglial marker, purinergic receptor P2Y12 (P2RY12), studies have shown that the microglia assume a dysfunctional, dystrophic form at advanced disease stage, characterized by a reduction in P2RY12 immunoreactivity alongside an increase in IBA-1 immunoreactivity within the spinal cord [93]. Similarly, microglia isolated during the symptomatic period from mSOD1 mice have been shown to exhibit features indicative of a SASP, displaying β-galactosidase activity, and elevated levels of p16, MMP-1, p53 and nitrotyrosine, accompanied by a large and flattened morphology [94].

In the Tau-P301S mice, a model related to AD, senescent microglial cells and astrocytes were observed at six months of age [95, 96]. In this study, two methods, genetic engineering and senolytic treatment, were used to eliminate senescent cells. Both approaches reduced tau pathology and gliosis, and prevented neuronal degeneration, resulting in improved cognitive function. This suggests that senescent glial cells directly contribute to neuronal tau pathology and cognitive impairment, making them a potential therapeutic target.

Relatively, there is limited direct evidence so far for the involvement of senescent microglia in Parkinson's disease (PD). α-Synuclein, the main protein that aggregates in PD, can also interact with microglia. In addition, aging triggers the transition of microglia from neuroprotective to senescent phenotype, along with an elevated concentration of senescent microglia in the substantia nigra [49]. Based on the previous studies indicating that overexpression of α-synuclein leads to cellular senescence [97], it can be speculated that persistent exposure to α-synuclein may induce glial cell senescence. Furthermore, the increased ferritin levels, a characteristic of senescent microglia, may be involved in PD, which has been epidemiologically linked to iron exposure [49]. However, further studies are needed to address this issue in PD.

## Driving forces of microglial senescence and their phenotype

### Genomic instability

Genomic instability refers to a state in which the genome is frequently mutated by DNA damage and DNA replication errors. This process leads to accumulation of DNA mutations that are either deleterious or silencing, resulting in a disease state or maintaining a physiological state [98]. While research on the impact of microglial DNA damage on neurodegenerative diseases such as ALS [99] and ataxia telangiectasia [100] is being actively conducted, there is a paucity of studies on its association with senescent microglia. Although reports of genomic instability in microglia are limited, given the microglial susceptibility to aging, particularly in white matter [50], and their limited capacity for repopulation [40], it is plausible to speculate that genomic instability in microglia could be influenced by both intrinsic characteristics and external environmental factors. These factors may include the accumulation of myelin debris, toxic protein aggregates, and various toxic molecules.

*Ercc1* participates in DNA damage repair. In *Ercc1*-deficient mice, microglia exhibit a reactive and accelerated aging phenotype characterized by a hypertrophic morphology, increased response to lipopolysaccharide (LPS), up-regulation of the inflammatory response and production of ROS [48]. In contrast to the effects observed in constitutive *Ercc1*-deficiency mice, the specific deletion of Ercc1 in microglia did not induce microglial activation or enhance their responsiveness to a systemic LPS challenge. Gene expression analysis showed that the deletion of *Ercc1* in microglia resulted in a transient aging signature [101]. In addition, double-strand breaks can lead to erosion of epigenetic information through DNA damage repair following chromatin reorganization and increase the number of primed microglia [102]. Impaired DNA damage repair can accelerate cellular senescence, leading to the accumulation of DNA damage and epigenetic loss [17]. These factors, in turn, may induce senescent microglia to release pro-aging factors (e.g. type-I IFN, etc.) [69, 103] into the environment. Thus, neuroinflammation may be a secondary consequence of cellular senescence and an amplification of cellular senescence as well as a primary driver of brain aging. These changes at the genomic and epigenomic levels can induce genotoxicity, leading to cell cycle arrest to protect the daughter cell from the transfer of damaged genetic information. To inhibit cell cycle progression, microglia express cyclin-dependent kinase inhibitors, including p16 and p53, which are senescence markers [104, 105].

### Cytoplasmic lipid droplets and protein aggregates

The term “proteostasis” refers to a state of balanced processes of protein translation, folding, maintenance, and degradation. In the aging brain, microglia also undergo a decline in proteostasis [106, 107]. Recent next-generation sequencing studies have demonstrated dysregulation of mRNA splicing, reduced expression of ribosomal proteins, discordant profiles of transcripts associated with mitochondrial function, and even epigenetic changes in the aging brain [108–110]. This evidence highlights the accumulation of misfolded proteins and the generation of dysfunctional proteins in the aging brain. Key proteins involved in this process include tau, Aβ, TDP-43, and α-synuclein, all of which are recognized contributors to neurodegeneration [111, 112].

Studies have demonstrated the critical role of normal autophagy and lysosomal acidification in promoting lifespan extension in *Caenorhabditis elegans* [113, 114]. Specifically, these processes play critical roles in cellular signaling, energy metabolism, and maintenance of a functional proteome [115]. It is important to investigate how impairment of lysosomal function contributes to the accumulation of intralysosomal granules such as lipid droplets, lipofuscin, and disease-associated protein aggregates. These granules may serve as alternative energy sources or drivers of cellular dysfunction in senescent microglia.

Recent studies have shown that lysosomal dysfunction, coupled with altered lipid metabolism, contributes to cellular senescence. Lipid droplets, acting as a lipid storage, play a role in energy homeostasis and interact with mitochondria and lysosomes [116]. Lipid droplets directly contact mitochondria via perilipin 5, providing fatty acids for beta-oxidation in situations of starvation [117]. Lipid droplets serve as an alternative energy source when cellular energy required for growth and maintenance of physiological functions is depleted. As cells age, mitochondrial energy production decreases, leading to an increase in intracellular lipid droplets as a compensatory mechanism for survival. However, the accumulation of lipid droplets can have detrimental effects on cells. For example, LDAM observed in the aging brain show decreased phagocytic activity and increased secretion of ROS and pro-inflammatory cytokines [63]. In addition, lipid-laden microglia in the ischemic brain of an aged mouse show impaired neuroprotection [118]. The cytoplasmic accumulation of ferritin is reduced in microglia, resulting in increased intracellular iron levels [119]. Furthermore, iron accumulation in microglia not only affects ferroptosis, but also impairs the phagocytic activity of these cells [120]. Iron metabolism in microglia is regulated by heme oxygenase-1 and SEC24B. Impaired iron metabolism contributes to microglial aging and neurodegeneration [121, 122].

As described above, the accumulation of lipid droplets, lipofuscin, SA-β-gal, and iron within microglia can lead to the hypophagocytic function of microglia and disrupt metabolism [123]. Accumulating granules and aggregated proteins can disrupt cellular homeostasis and increase cellular complexity, thereby contributing to senescence.

### Bioenergetics

The metabolic environment of the brain is unique compared to other organs, meeting the high metabolic demands of neuronal activity and facilitating nutrient transport across the BBB [124]. Furthermore, dysfunction of mitochondria, the hub of cellular energy metabolism, is recognized as a primary pro-aging feature [125]. Consequently, there is a growing interest among researchers on bioenergetics and its impact on brain aging. A growing body of evidence supports the notion that senescent cells have altered lipid metabolism [24]. For example, human fibroblasts undergoing oncogene-induced senescence show increased β-oxidation activity, which is the process of lipid metabolism [126]. However, most studies on the metabolism of senescent cells have been conducted in fibroblasts and cell lines, rather than in microglia. One study shows that long-lived individuals have increased β-oxidation [127], suggesting that enhancing β-oxidation may promote longevity, since it typically declines with age.

The aerobic glycolysis pathway is commonly used for rapid energy production, and in myeloid cells, including microglia, a shift to this pathway is associated with inflammatory activation [128]. Microglia in the inflammatory state show increased lactate production and decreased mitochondrial oxygen consumption, leading to an augmented reliance on glycolysis [129, 130]. Considering that microglia are innate immune cells, rapid ATP production via glycolysis seems to be a reasonable response for proper immune actions and cytokine release as well as phagocytic function. In physiological and healthy states, microglia prefer oxidative phosphorylation (OXPHOS), a process involving mitochondria-mediated production of large amounts of ATP [131]. Specifically, during senescence, microglial glucose metabolism shifts from OXPHOS to glycolysis, a process regulated by hexokinase 2 and 6-phosphofructro-2-kinase 3 [132, 133]. These metabolic shifts are associated with an increase in Hif-1α (hypoxia-inducible factor 1 alpha) and the mTOR pathway, which has been implicated in the regulation of cytokine secretion and phagocytosis in microglia [134].

Excessive lipids play an important role in cellular senescence [127], with fatty acids directly or indirectly regulating microglial phagocytosis, cytokine secretion, and immune surveillance. The TREM2–APOE axis primarily controls lipid metabolism in microglia. TREM2, which senses lipids, is involved in myelin phagocytosis, and excessive myelin debris could lead to the accumulation of cholesteryl esters, major components of lipid droplets [60, 135]. Microglial ATG7, a key regulator of the autophagy-lysosomal pathway, plays a role in regulating lipid metabolism [136]. It has been suggested that the age-related decline in lysosomal function may lead to dysregulation of lipid metabolism, contributing to the induction of lipid droplets. Therefore, the accumulation of lipid droplets in senescent microglia can be attributed to various factors, including excessive lipid intake, impaired fatty acid metabolism leading to increased reliance on glycolysis, or genetic factors such as *Grn* mutations.

As a result, research on the metabolic profile of microglia in brain aging has primarily focused on inflammatory metabolic changes [137, 138]. However, given the multifaceted nature of microglia, it is critical to examine their metabolism and phagocytic function beyond their inflammatory cues. At the forefront of aging, microglia play a central role in low-grade neuroinflammation by recognizing and dealing with cellular debris such as lipid droplets and accumulated iron, a concept termed “Garb-aging” [139]. As a result, senescent microglia, which exhibit impaired phagocytosis and metabolic dysfunction, may contribute to the accumulation of myelin debris, protein aggregates and toxic substances, ultimately contributing to the processes of aging and the development of neurodegenerative diseases [52].

**Fig. 3:**
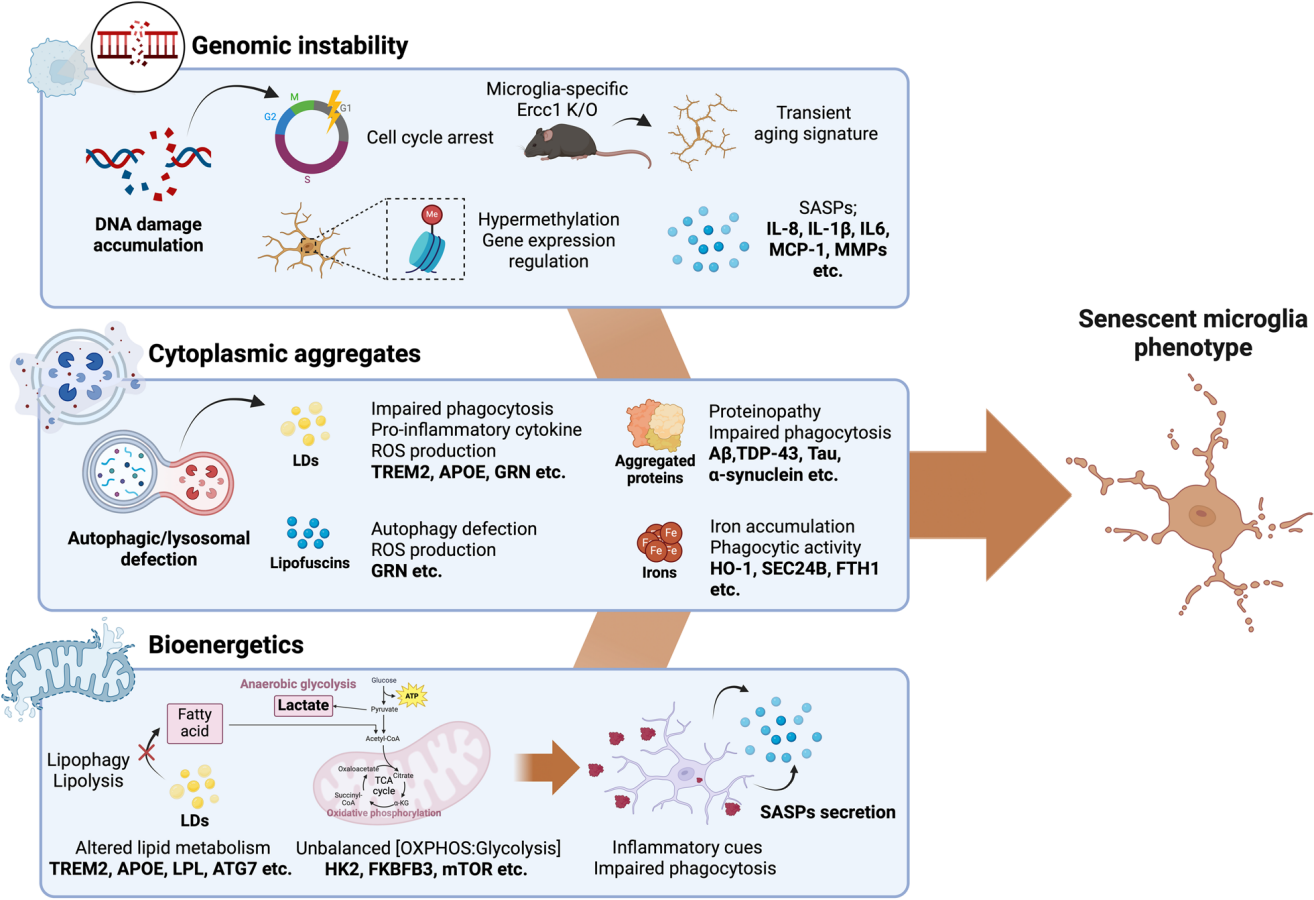
Driving forces and phenotypes of senescent microglia. (1) Genomic instability: Accumulating DNA damage arrests the cell cycle and regulates gene expression through epigenetic modifications. As a result, secretion of a senescence-associated secretory phenotype is increased, leading to a primed microglial response. (2) Cytoplasmic aggregates: Autophagy or lysosomal dysfunction can lead to abnormal protein degradation or inability to degrade excess lipids, resulting in impaired phagocytosis. These defects can lead to intracellular iron accumulation and either microglial senescence or ferroptosis-resistance. (3) Bioenergetics: Chronic phagocytic challenges due to either disease-associated protein aggregates or excessive myelin debris can lead to alterations in mitochondrial metabolism and energy sources, as well as alterations in the TREM2-ApoE axis. Created with Biorender.com

In summary, the increased granularity of microglia contributes to their impaired phagocytosis and dysregulated metabolism, creating a vicious cycle of accelerated brain aging. Given the active interactions between microglia and other brain cells, including neurons, it is imperative to recognize that the metabolically reprogrammed microglia with accumulation of lipid droplets, lipofuscins, and protein aggregates could affect CNS energy metabolism, potentially disrupting cellular homeostasis and initiating the process of inflammaging. The overall concept is illustrated in Fig. 3.

## Rejuvenating senescent microglia: conquering neurodegenerative diseases

In light of recent reports indicating that senescent microglia are a common pathological feature of neurodegenerative diseases, there has been a growing effort to apply known senolytics to these conditions [140, 141]. Senolytics are compounds that target and eliminate senescent cells through a process called senolysis, reducing the burden of senescent cells and improving the lifespan of the organism. For example, injection of dasatinib with quercetin (D + Q), repurposed drugs used as senolytics, has been shown to reduce senescent cells and attenuate inflammation in human adipose tissue [142]. In addition, whole-body clearance of p16-ATTAC-positive senescent cells in a transgenic mouse model with AP20187 can specifically eliminate senescent microglia and reverse cognitive decline [143, 144].

Elimination of the p16^INK4A^-positive senescent cells in BubR1 progeroid mice by activation of the INK-ATTAC transgene significantly delays age-related diseases [145]. In mice with age-related AD, senolytic treatment selectively removes senescent cells from the amyloid plaque environment, reduces neuroinflammation, decreases Aβ burden, and ameliorates cognitive deficits [146]. Although current senolytics are not specifically designed to target senescent microglia in the brain, results of initial clinical trials for AD raise hope for the potential use of senolytics in the treatment of neurodegenerative diseases [140].

Several studies have investigated the rejuvenation of the aging brain using biological factors from young organisms. Young blood, human umbilical cord plasma, and young cerebrospinal fluid (CSF) can manipulate the plasma and CSF compositions in older mice via pro-youthful factors, leading to brain rejuvenation, although the therapeutic purpose was not to specifically target senescent microglia [103]. Exposure to old blood, either through heterochronic parabiosis or administration of old blood plasma, results in decreased hippocampal neurogenesis and synaptic plasticity, increased microgliosis, and impaired learning and memory via pro-aging factors such as CCL2 and CCL11 [147]. Conversely, exposure of aged mice to young blood enhances hippocampal neurogenesis, increases dendritic spine density, and improves learning and memory [148]. Intravenously injected human umbilical cord plasma proteins can improve cognitive function and revitalize hippocampal neurons via TIMP2 (tissue inhibitor of metalloproteinases 2) [149]. In addition, injection of young CSF into aged mice can restore memory, with increased proliferation of oligodendrocyte precursor cells [150]. While these strategies have primarily been studied for their effects on age-related brain functions, they may be useful as potential methods for microglial rejuvenation.

The central idea behind microglial rejuvenation is to transform senescent microglia into healthy or youthful microglia. Several approaches are being explored for microglial rejuvenation, including functional restoration, metabolic reprogramming, and anti-inflammaging. Senescent microglia often exhibit impaired phagocytic activity [6]. Restoration of phagocytosis in senescent microglia has been achieved by various means, including inhibition of the CD22 receptor, which is known for its interaction with low-density lipoprotein [151]. In addition, the phagocytic function can be restored by regulating lipoprotein lipase through inhibition of hexokinase 2, a critical enzyme in glucose metabolism [152]. Overexpression of CD36, also known as fatty acid translocase, improves the remyelinating function of microglia in aged brain [153]. Genes responsible for the regulation of phagocytosis and remyelination in senescent microglia are closely linked to lipid metabolism, suggesting that reprogramming metabolism may be a key aspect for microglial rejuvenation [60].

Although it has not been confirmed whether metabolic reprogramming alone can rejuvenate senescent microglia, recent studies show that energy metabolism could at least restore the impaired microglial phagocytic function. A recent study highlighted the potential of pharmacological inhibition of EP2/4, receptors for PGE_2_, to rejuvenate energy metabolism in energy-deficient microglia and myeloid cells [154]. In this process, glycogen is sequestered into glucose by GYS1 [154]. Given that prostaglandin synthesis plays a critical role in reinforcing the cell cycle arrest associated with senescence [155], the EP2/4 pathway may be a potential target for rejuvenating senescent microglia.

In addition, the antibody transport vehicle (ATV) system, which is capable of crossing the BBB, has been used to generate a recombinant TREM2-activating antibody [156]. This antibody enhances glucose metabolism through both the mTOR and the PLCG2 pathways, making it a promising approach for AD. However, we cannot exclude the possibility that the recombinant anti-TREM2 antibody may increase senescent microglia in AD [61]. The cGAS-STING pathway, known for its role in immune sensing of viral DNA or cytosolic DNA, plays a critical role in the initiation of inflammation. Given its function as a damage-associated molecular pattern signal, the cGAS-STING pathway may be associated with inflammaging [157]. The STING inhibitor H-151, as an anti-inflammtory compound, was found to reverse cognitive impairment and alleviate neuroinflammation in microglia of the aged brain [158].

**Fig. 4:**
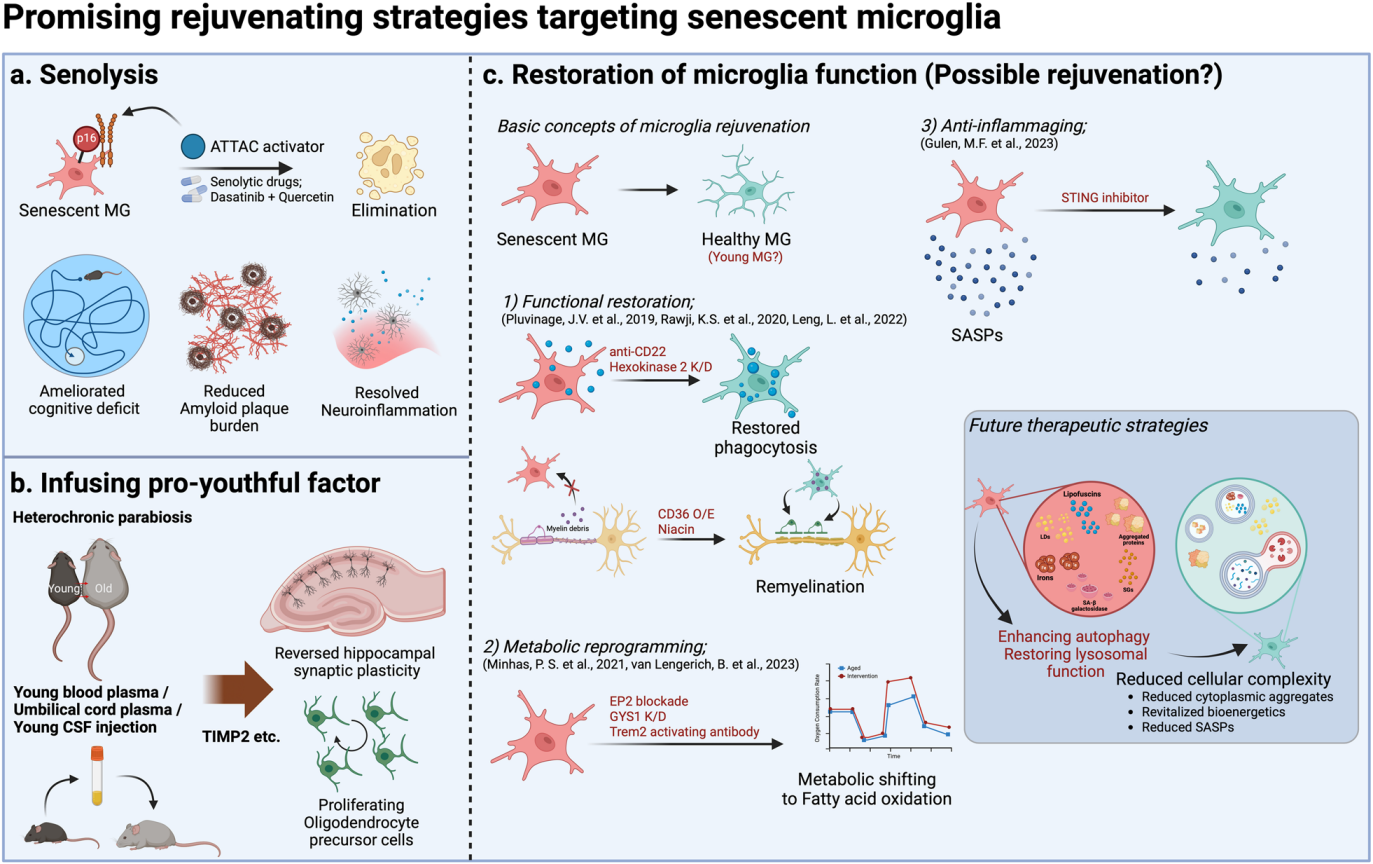
Promising strategies for microglia-targeted senotherapy. **a** Senolysis: Healthy and senescent microglia coexist in the aging brain, and senolysis targets and removes only the senescent microglia. **b** Infusion of youthful factor: To restore cognitive function in old mice, cerebrospinal fluid or plasma from young mice was administered. Further research is needed to determine which factors in the young mice reverse aging. **c** Restoration of microglial function: Microglial rejuvenation can be achieved by restoring the function of senescent microglia, revitalizing metabolism, and inhibiting inflammaging. Further research is needed. Created with Biorender.com

The promising approach of enhancing autophagy and restoring lysosomal function is gaining attention. One study highlights how autophagy enables microglia to interact with amyloid plaques and prevents microglial senescence [68]. It showed that inhibition of microglia-specific autophagy exacerbates neuropathology in AD mice, leading to the development of senescent microglia characterized by p21 expression, dystrophic morphologies, and SASP. However, in this study, the authors used classical senolytics (D + Q) to eliminate senescent microglia rather than a strategy to restore autophagy in these cells. Therefore, rejuvenation of senescent microglia by methods such as metabolic reprogramming, autophagy restoration, and senolytics may represent an alternative treatment strategy for neurodegenerative diseases. The possible rejuvenation strategies targeting senescent microglia are summarized in Fig. 4.

## Conclusion and future prospects

In contrast to the aging process observed in healthy brains, the increased prevalence of age-related neurodegenerative diseases appears to be intricately linked to an accelerated form of brain aging. This accelerated trajectory of brain aging is thought to be influenced by factors such as genetic instability, abnormal protein aggregates, compromised mechanisms for clearance of these aggregates, impaired mitochondria function, and inflammaging. Notably, senescent microglia are located in regions susceptible to brain aging, highlighting their potential role as a focal point of this accelerated brain aging.

These novel insights hold promise for illuminating the underlying pathophysiology of brain aging and its intricate relationships with neurodegenerative diseases. Consequently, strategies that take a more nuanced approach and target the elimination or rejuvenation of senescent microglia within the realm of senotherapy, as documented to date, are emerging as highly encouraging avenues. These avenues offer the potential for the advancement of specialized therapeutic techniques uniquely designed to effectively address the complex issue of neurodegenerative diseases associated with brain aging.

## Data Availability

Not applicable.

## Abbreviations

AD: Alzheimer’s disease
ALS: Amyotrophic lateral sclerosis
APOE: Apolipoprotein E
OXPHOS: Oxidative phosphorylation
SASP: Senescence-associated secretory phenotype
ROS: Reactive oxygen species
LDAM: Lipid droplet-accumulating microglia
MGnD: Microglial neurodegenerative phenotype
DAM: Disease-associated microglial phenotype
IFN-γ: Interferon-gamma
IL: Interleukin
LPS: Lipopolysaccharide
mTOR: Mammalian target of rapamycin
MMP: Matrix metalloproteinase
mSOD1: Mutant superoxide dismutase 1
PGE_2_: Prostaglandin E2
P2RY12: Purinergic receptor P2Y
SA-β-gal: Senescence-associated beta-galactosidase
TDP-43: TAR DNA-binding protein 43
TREM2: Triggering receptor expressed on myeloid cells 2
BBB: Blood brain barrier
